# Large German Multicenter Experience on the Treatment Outcome of 207 Patients With Adenoid Cystic Carcinoma of the Major Salivary Glands****

**DOI:** 10.3389/fonc.2020.593379

**Published:** 2020-11-11

**Authors:** Sati Akbaba, Tilman Bostel, Kristin Lang, Suzan Bahadir, Djoeri Lipman, Heinz Schmidberger, Christoph Matthias, Nicole Rotter, Andreas Knopf, Christian Freudlsperger, Peter Plinkert, Juergen Debus, Sebastian Adeberg

**Affiliations:** ^1^ Department of Radiation Oncology, University Medical Center Mainz, Mainz, Germany; ^2^ Department of Radiation Oncology, University Hospital Heidelberg, Heidelberg, Germany; ^3^ Department of Radiology, University Hospital Heidelberg, Heidelberg, Germany; ^4^ Department of Radiology, Koru Hospitals-Yuksek Ihtisas University, Ankara, Turkey; ^5^ Department of Radiation Oncology, Isala Hospital Zwolle, Zwolle, Netherlands; ^6^ Department of Laryngology and Head and Neck Surgery, University Medical Center Mainz, Mainz, Germany; ^7^ Department of Laryngology and Head and Neck Surgery, University Hospital Mannheim, Mannheim, Germany; ^8^ Department of Laryngology and Head and Neck Surgery, University Hospital Freiburg, Freiburg im Breisgau, Germany; ^9^ Department of Oral and Maxillofacial Surgery, University Hospital Heidelberg, Heidelberg, Germany; ^10^ Department of Laryngology and Head and Neck Surgery, University Hospital Heidelberg, Heidelberg, Germany

**Keywords:** multicenter study, carbon ion radiotherapy, adenoid cystic carcinoma, major salivary glands, local control, recurrence patterns, toxicity

## Abstract

**Introdution:**

We aimed to evaluate treatment outcome of combined radiotherapy (RT) including photon intensity modulated radiotherapy (IMRT) and carbon ion boost for adenoid cystic carcinomas (ACCs) of the major salivary glands, the currently available largest German collective for this cohort.

**Materials and Methods:**

Overall, 207 patients who were irradiated with combined RT between 2009 and 2019 at Heidelberg University Hospital were analyzed retrospectively for local control (LC), progression-free survival (PFS) and overall survival (OS) using Kaplan-Meier estimates. The majority of patients received postoperative RT (n=176/207, 85%) after previous surgery in large German hospitals mainly Mainz, Freiburg, Mannheim and Heidelberg University Hospitals and 15% received primary RT (n=31/207).

**Results:**

After a median follow-up time of 50 months, 84% of the patients were still alive (n=174/207). Disease progression occurred in 32% of the patients (n=66/207) while local recurrence was diagnosed in 12% (n=25/207), and distant relapse in 27% (n=56/207). Estimated 5-year LC, PFS and OS rates were 84%, 56% and 83% for OS, respectively. In multivariate analysis, we could identify two prognostic subgroups: one subgroup resulting in decreased LC, PFS and OS rates and another subgroup having an additional survival disadvantage in PFS and OS. Patients with a macroscopic tumor disease (yes vs. no; p<0.001 for LC, p=0.010 for PFS and p=0.040 for OS) treated in a definitive setting (vs. postoperative setting; p=0.001 for LC, p=0.006 for PFS, p=0.049 for OS) and tumors of upper T stage (T1-4; p=0.004 for LC, p<0.001 for PFS, p<0.001 for OS) showed significantly more local relapses and a decreased PFS and OS. Upper Age (p<0.001 for both PFS and OS), lower Karnofsky Performance Score (<80% vs. ≥80%; p<0.001 for both PFS and OS) and solid histology (vs. non-solid; p=0.049 for PFS and p=0.003 for OS) were in addition associated with worse survival outcome. Toxicity was moderate with 18% late grade 2 and 3 toxicity.

**Conclusions:**

Combined RT results in superior LC rates compared to photon data with moderate toxicity. In multivariate analysis, upper T stage, the existence of a macroscopic tumor before RT and definitive RT setting were identified as major prognostic factors affecting LC negatively.

## Introduction

Three of four tumors arising from the major salivary glands are benign while only one of four is from malign nature (1–3). The World Health Organization (WHO) classification system differentiates 25 distinct malignant salivary gland tumor types. Adenoid cystic carcinoma (ACC) is the second most common malign salivary gland tumor in the parotid gland and the most common malign subtype in the submandibular and sublingual gland (4–6). Overall, ACCs correspond to less than 0.15% of all malignancies in the head and neck (7–9).

Two histological forms of ACC are distinguished (10). The cribriform and the tubular subtypes originate from epithelial and myoepithelial cells. The solid form only consists of either the epithelial component by loss of the myoepithelial component or the myoepithelial component by loss of the epithelial component. Solid ACCs are known to be more aggressive with more frequently developing local relapses and distant failure. Due to their histological diversity no grading system for ACC exists.

ACCs are characterized by a slow but locally aggressive growth and a high rate of distant metastases even years after first diagnosis resulting in a poor prognosis (1, 11, 12). Up to 70% of recurrences are diagnosed within 3 years of first diagnosis and nearly 50% of patients develop distant metastases during follow-up predominantly involving the lungs (up to 80%) followed by the bone, liver and other sites (13, 14).

Standard treatment consists of primary complete surgical excision and postoperative radiotherapy (RT) for advanced stages (T3/4, N+) or incomplete resection margins (R1/2). Nevertheless, several studies show that all tumor stages (T1-4) profit from postoperative RT (11, 13, 15–17). To date, international guidelines for treatment of salivary gland tumors are lacking. In some cases, primary RT is indicated for inoperable tumors (18, 19). ACCs are known to be relatively radio resistant that high RT doses over 80 Gy are necessary to achieve local control. Indeed, dose escalation is limited by several surrounding organs, e.g. optic system, inner ear and skull base (11, 20, 21).

Several pre- and post-treatment prognostic factors affecting local control (LC), progression-free survival (PFS) and overall survival (OS) are identified within the last decades (3, 22, 23). The development of more modern RT techniques, e.g. intensity modulated radiotherapy (IMRT) and carbon ion RT (CIRT), queries the impact of known prognostic factors on local control and survival.

The current study analyzes 207 ACCs of the major salivary glands (parotid gland, submandibular gland and sublingual gland) treated with modern RT combining IMRT and a CIRT boost to the macroscopic tumor for definitively and to the tumor bed for postoperatively treated patients. Therefore, medical records from the cancer registry of Heidelberg University Hospital and the external medical reports from the respective reference centers were studied. In multivariate analysis, prognostic factors for this treatment regime were identified which differed partially from previously known factors. In addition, toxicity according to the Common Toxicity Terminology Criteria for Adverse Events (CTCAE) v5 was assessed.

## Materials and Methods

### Patient Selection

The medical records of patients with ACC of the major salivary glands (parotid gland, submandibular gland and sublingual gland), who were treated between 2009 and 2019 with bimodal RT including IMRT and CIRT and received regular follow-up examinations, were analyzed retrospectively. Overall, 207 consecutive patients were identified. Patient, tumor and treatment characteristics were assessed from the cancer registry of Heidelberg University Hospital and the external medical reports from the respective reference centers where the first diagnosis was made. The majority of patients were transferred to Heidelberg University Hospital predominantly from large German centers mainly Mainz, Freiburg and Mannheim University Hospitals. Pathology reports were studied for perineural invasion (PNI, yes vs. no), resection margins (R0 vs. R1 vs. R2) and histology (solid vs. non-solid) while only tumors with more than 30% solid parts were considered as solid. TNM stage at first diagnosis was based on initial radiologic imaging (computed tomography (CT) and magnetic resonance imaging (MRI)) in case of definitively treated patients and additionally on pathologic reports for postoperatively irradiated patients. The presence of a macroscopic tumor (yes vs. no) before RT start was assessed with current MRIs before treatment start for all patients. This analysis has been approved by the independent ethics committee of the medical faculty Heidelberg.

### Procedures

A CT scan in head-first supine position with a thermoplastic head mask for immobilization was performed before RT start for each patient. A current MRI and in case of postoperative RT an additional preoperative MRI was matched to the CT scan in irradiation position in form of a rigid co-registration for tumor demarcation and target delineation. A gross tumor volume (GTV) was defined for macroscopic tumor based on the current MRI. Two different clinical target volumes (CTVs) were outlined. CTV1 included the macroscopic tumor (GTV) or tumor bed (if no GTV was available) with a safety margin of 5 mm and CTV2 included CTV1 and typical local and regional pathways of tumor spread. For parotid gland ACCs, the ipsilateral cervical lymph node levels I, II, III, and IVa and for submandibular and sublingual ACCs, the bilateral cervical lymph node levels I, II, III, and IVa were included into the CTV2. For uncertainties in patient positioning, planning target volumes (PTVs) were created. Therefore, an isotropic margin of 3 mm was added to the CTVs. The 3 mm margin was reduced at sites where the tumor infiltrated critical structures.

Patients were followed up every 3 months until 2 years after RT, every 6 months until 3 years after RT and then annually until 10 years after RT with MRI and an annually performed CT of the chest and upper abdomen to exclude tumor spread into the lungs, bone and liver. Patients were either seen at the Heidelberg University Hospital for radiologic and clinical examination during follow-up or at the reference centers. If regular follow-up examinations were wished at the reference centers by the patient, external clinical reports and current radiologic images were regularly sent to Heidelberg for second opinion during follow-up. Toxicity was assessed according to CTCAE v5. Acute toxicity was defined as any adverse event during RT up to six weeks after RT and late toxicity as any adverse event six weeks after RT or later up to the last follow-up examination.

### Treatment and Treatment Planning

For treatment planning Syngo PT PlanningVersion 13 (Siemens, Erlangen, Germany) was applied for CIRT and TomoTherapy^®^-Planning Station (Accuray, Sunnyvale, CA, USA) for photon RT (24). Carbon ion boost to the PTV1 was performed with 18–24 Gy in 3.0 Gy (RBE, relative biological effectiveness) fractions (five to six fractions per week) and photon RT to the PTV2 was performed with 46 Gy to 56 Gy in 2.0 Gy fractions (five fractions per week) for both postoperative and definitive RT due to the local aggressiveness and radioresistance of ACC. Median total equivalent dose to 2.0 Gy fractions (EQD2) prescribed to the median PTV and covering the 95% prescription isodose was 80 Gy (range 71–82 Gy).

**Figure 1:**
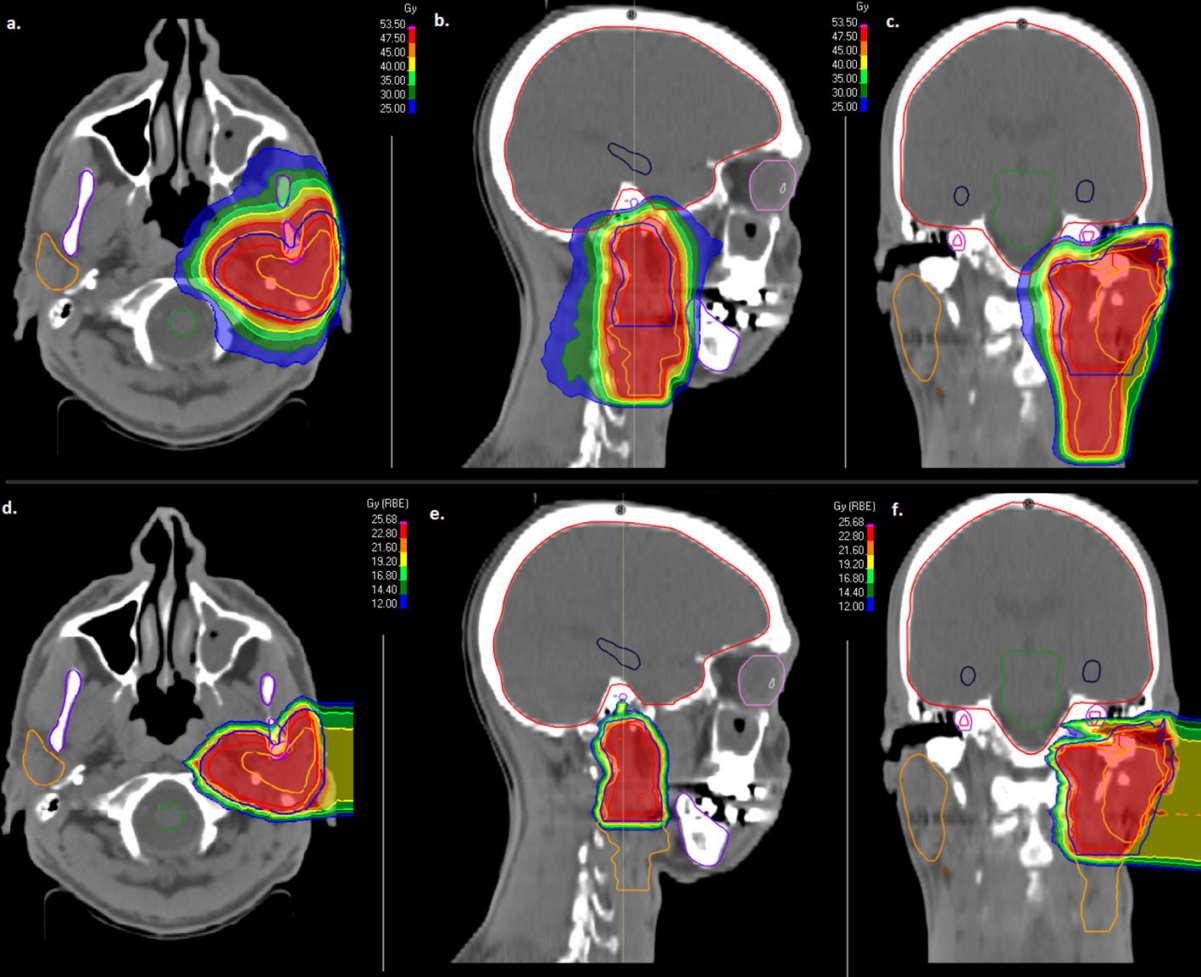
Combined treatment plan with intensity modulated radiotherapy (IMRT, **A–C**) and active raster-scanned carbon ion radiotherapy (CIRT, **D–F**) of a patient with a pT3pN0 (0/34) ACC of the left parotid gland. CTV2 involved the left parotid space and the left cervical lymphatic drainage **(A–C)** and CTV1 involved the left parotid space only **(D–F)**. IMRT was applied with 50 Gy in 2 Gy fractions **(A–C)** and CIRT was applied with two beams up to 24 Gy (RBE) in 3 Gy (RBE) fractions **(D–F)**. From left to right, axial dose distribution, sagittal dose distribution and coronal dose distribution are depicted.

Delivered doses to critical structures were limited according to current guidelines as low as possible in order to reduce RT-related toxicity (**Supplementary Table 1**) (25, 26). In **Table 1**, treatment characteristics are shown in detail. In **Figure 1**, the combined treatment plan for a patient with a pT3pN0 (0/34) ACC of the left parotid gland is shown. CTV2 involved the left parotid space and the left cervical lymphatic drainage (**Figures 1A–C**) and CTV1 involved the left parotid space only (**Figures 1D–F**).

**Table 1:** Patient, tumor and treatment characteristics (n= 207).

Characteristic	Overall (n=207)	Definitive RT (n=31, 15%)	Postoperative RT (n=176, 85%)	p-values
Age, years				0.141
median age	53	52	60	
range	12–86	12–86	25–83	
Gender, no. (%)				0.241
male	83 (40)	11 (35)	72 (41)	
female	124 (60)	20 (65)	104 (59)	
KPS, no. (%)				0.474
≥80%	188 (91)	28 (90)	160 (91)	
<80%	19 (9)	3 (10)	16 (9)	
Tumor site, no. (%)				0.076
parotid gland	108 (52)	21 (67)	87 (49)	
submandibular gland	82 (40)	7 (23)	75 (43)	
sublingual gland	17 (8)	3 (10)	14 (8)	
Initial TNM, no. (%)				
T1	25 (12)	2 (6)	23 (13)	***0.012***
T2	46 (22)	3 (10)	43 (24)	
T3	70 (34)	3 (10)	67 (38)	
T4	50 (24)	18 (58)	32 (18)	
Tx	16 (8)	5 (16)	11 (6)	
N0	139 (67)	18 (58)	121 (69)	0.504
N+	54 (26)	8 (26)	46 (26)	
Nx	14 (7)	5 (16)	9 (5)	
M0	188 (91)	21 (68)	167 (95)	**<0.001**
M1	19 (9)	10 (32)	9 (5)	
Histology, no. (%)				0.539
solid	113 (55)	14 (45)	99 (56)	
non-solid	68 (33)	10 (32)	58 (33)	
unknown	26 (13)	7 (23)	19 (11)	
PNI, no. (%)				***0.003***
yes	138 (67)	10 (33)	128 (73)	
no	43 (21)	14 (45)	29 (16)	
unknown	26 (13)	7 (23)	19 (11)	
Setting of RT, no. (%)				0.040
primary	177 (86)	19 (61)	158 (90)	
at recurrence	30 (14)	12 (39)	18 (10)	
Resection margin, no. (%)				NA
R0	43 (21)	─	43 (24)	
R1	99 (48)	─	99 (56)	
R2	13 (6)	─	13 (7)	
Rx	22 (11)	─	22 (13)	
Treatment dose, EQD2 in Gy				0.480
median	80	80	80	
range	71-82	71-82	73-81	
CTV1, ccm				0.128
median	81	160	96	
range	20-437	28-437	20-414	
CTV2, ccm				0.054
median	292	390	275	
range	112-977	121-977	112-806	

KPS, Karnofsky Performance Score; PNI, perineural invasion; EQD2, equivalent dose to 2 Gy fractions; CTV1, clinical target volume 1 including the tumor/tumor bed (Boost volume); CTV2, clinical target volume including CTV1 and neck lymph nodes.

### Statistical Analysis

Local control (LC, time from end of RT to the first appearance of local relapse or last follow-up), progression-free survival (PFS, time from first diagnosis to the first appearance of local relapse, distant relapse, death from any cause or last follow-up) and overall survival (OS, time from first diagnosis to death from any cause or last follow-up) were calculated using Kaplan-Meier estimates. In univariate analysis, the correlation of age, gender, Karnofsky Performance Score (KPS, <80% vs. ≥80%), tumor site (parotid gland vs. submandibular gland vs. sublingual gland), T stage (T1-4), N stage (N0 vs. N+), histology (solid vs. non-solid), perineural invasion (PNI, yes vs. no), treatment modality (definitive vs. postoperative), resection margins (R0 vs. R1 vs. R2), macroscopic tumor before RT start (yes vs. no) and CTV (<120 ccm vs. ≥120 ccm) with all three time-to-event data LC, PFS and OS were tested and potential prognostic factors were identified. For multivariate analysis, the cox-regression model was used. The Fisher’s Exact Test was used for categorial and the Wilcoxon signed-rank test for continuous data in order to compare patient and treatment characteristics for definitive vs. postoperative RT. Only p-values <0.05 were classified statistically significant. R version 3.4.2. (www.r-project.org) and SPSS Statistics version 24 (IBM, Armonk, New York, USA) were used for statistical tests.

## Results

### Patient Characteristics

Overall, 207 patients with ACC of the major salivary glands (52% parotid gland (n=108/207), 40% submandibular gland (n=82/207) and 8% sublingual gland (n=17/207)) were identified and included into statistical analysis. Patients received either definitive RT without any previous surgery (15%, n=31/207) or postoperative bimodal RT (85%, n=176/207). Within the definitive RT group, 12 patients were irradiated definitely for an inoperable macroscopic recurrence (39%) while 18 patients in the postoperative RT group were irradiated postoperatively for a local disease recurrence after previous treatment (10%). The majority of patients had tumors in advanced stages (58% T3/4, n=120/207). Macroscopic tumor before RT begin could be identified in 31% (n=65/207) on the basis of a pretreatment MRI. Patient and tumor characteristics for the whole cohort, for definitive bimodal and postoperative bimodal RT are presented in **Table 1**. Additionally, characteristics between the two treatment groups of definitive and postoperative bimodal RT were statistically compared (**Table 1**).

### Statistical Results

Median follow-up was 50 months (range 3–121 months). Overall, 12% local relapses (n=25/207), 32% disease progressions (n=66/207) and 16% deaths (n=33/207) were observed. Median time to local relapse, disease progression and death were 46 months for local relapse, 22 months for disease progression and 32 months for death. At last follow-up, 62% of the patients were alive without any progression, neither local nor distant (n=128/207).

**Figure 2:**
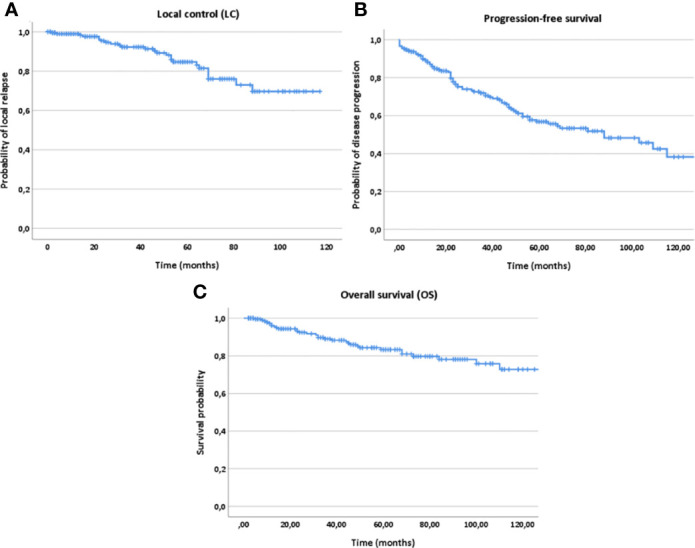
Kaplan-Meier estimates for local control **(A)**, progression-free survival **(B)** and overall survival **(C)** for all patients (n=207).

Estimated 5- and 10-year LC, PFS and OS rates for the whole patient collective were 84% and 69% for LC, 56% and 38% for PFS and 82% and 73% for OS, respectively (**Figure 2**). For postoperatively vs. definitely treated patients, the 5-year LC, PFS and OS rates were 88% vs. 61% for LC, 61% vs. 35% for PFS and 87% vs. 73% for OS. Regarding the 10-year outcome, these rates corresponded to 75% vs. 49% for LC, 39% vs. 29% for PFS and 76% vs. 61% for OS, respectively.

**Figure 3:**
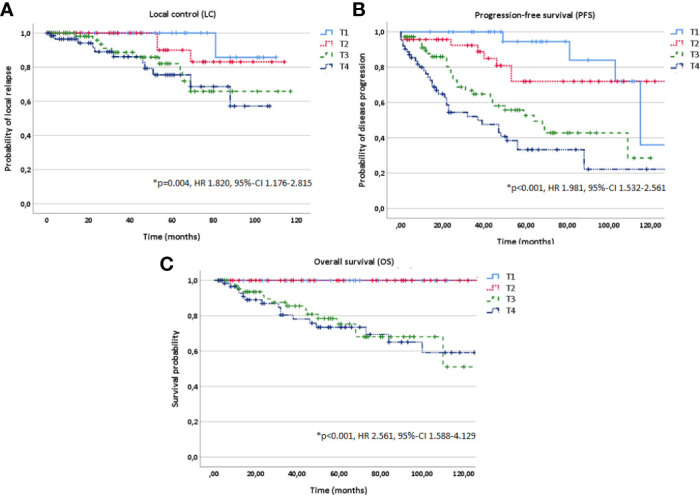
Kaplan-Meier estimates for local control **(A)**, progression-free survival **(B)** and overall survival **(C)** in dependence of T stage (T1-4).

In multivariate analysis tested for potential prognostic factors for LC, PFS and OS, T stage (T1-4), treatment modality (postoperative vs. definitive RT) and macroscopic tumor (yes vs. no) in a pretreatment MRI before RT were identified as independent prognostic factors for all three time-to-event data LC, PFS and OS (**Figures 3** **–** **5**, **Table 2**). Regarding the correlation between T stage and LC, 5-year LC was 100% for T1, 91% for T2, 82% for T3 and 75% for T4 (HR 1.820, 95%-CI 1.176–2.815, *p=0.004*). Patients treated with postoperative RT showed favorable LC rates with a 5-year LC of 87% compared to definitive RT with 60% (HR 3.813, 95%-CI 1.686–8.645, p=*0.001*) while the presence of a macroscopic tumor before RT resulted in decreased LC rates with a 5-year LC rate of 75% for macroscopic tumor vs. 93% for no macroscopic tumor (HR 6.548, 95%-CI 2.442–17.563, *p<0.001*). Tumor site (parotid gland vs. submandibular gland vs. sublingual gland, HR 0.654, 95%-CI 0.320–1.339, *p=0.246*), PNI (yes vs. no, HR 1.220, 95%-CI 0.474–2.736, *p=0.772*) and resection margins (R0 vs. R1 vs. R2, HR 1.071, 95%-CI 0.438–2.617, *p=0.880*) had no statistically significant impact on LC.

**Figure 4:**
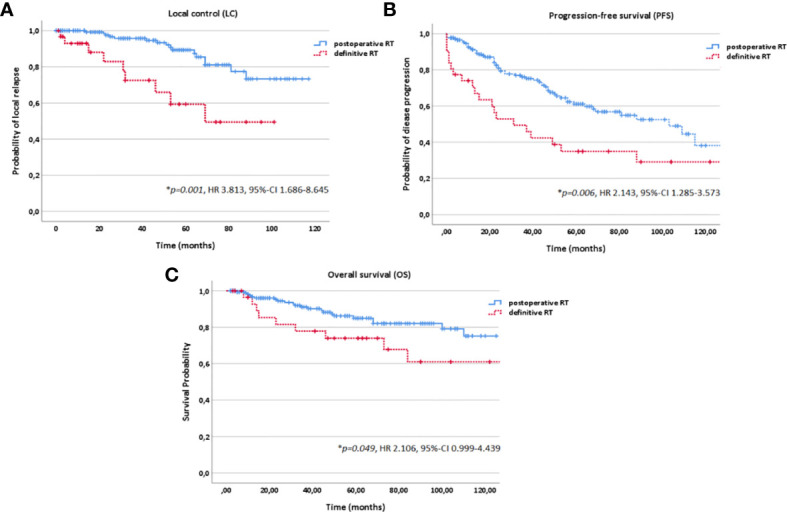
Kaplan-Meier estimates for local control **(A)**, progression-free survival **(B)** and overall survival **(C)** in dependence of the treatment modality (postoperative vs. definitive RT).

**Figure 5:**
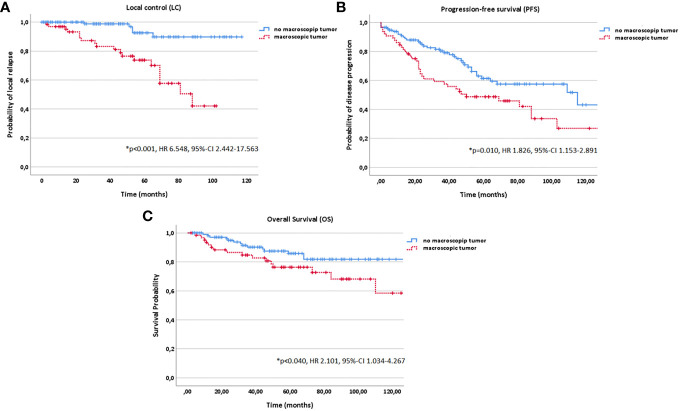
Kaplan-Meier estimates for local control **(A)**, progression-free survival **(B)** and overall survival **(C)** in dependence of macroscopic tumor diagnosed in a pretreatment magnetic resonance imaging (MRI) before radiotherapy (RT) start (yes vs. no).

**Table 2:** Results of multivariate analysis*.

*Characteristic*	*HR (95%-CI)*	*p-value*
**Local control (LC)**		
primary vs. postoperative RT	3.813 (1.686–8.645)	<0.001
T stage, T4 vs. T3 vs. T2 vs. T1	1.820 (1.176–2.815)	0.004
macroscopic tumor, yes vs. no	6.548 (2.442–17.563)	<0.001
CTV ≥120 ccm vs. <120 ccm	1.010 (0.890–1.112)	0.123
**Progression-free survival (PFS)**		
***Characteristic***	***HR (95%-CI)***	***p-value***
primary vs. postoperative RT	2.143 (1.285–3.573)	0.006
T stage, T4 vs. T3 vs. T2 vs. T1	1.981 (1.532–2.561)	<0.001
macroscopic tumor, yes vs. no	1.826 (1.153–2.891)	0.010
age (10 year steps)	1.311 (1.134–1.517)	<0.001
KPS, <80% vs.≥80%	3.542 (2.010–6.240)	<0.001
N stage, N+ vs. N0	1.244 (0.756–10.149)	0.286
**Overall survival (OS)**		
***Characteristic***	***HR (95%-CI)***	***p-value***
primary vs. postoperative RT	2.106 (0.999–4.439)	0.049
T stage, T4 vs. T3 vs. T2 vs. T1	2.561 (1.588–4.129)	<0.001
macroscopic tumor, yes vs. no	2.101 (1.034–4.267)	0.040
age (10 year steps)	1.963 (1.515–2.542)	<0.001
KPS, <80% vs.≥80%	6.472 (3.036–13.799)	<0.001
histology, solid vs. non-solid	2.808 (1.367–5.770)	0.003
progression, yes vs. no	89.553 (5.135–156.889)	0.002

HR, hazard ratio; CI, confidence interval; RT, radiotherapy; CTV, clinical target volume; KPS, Karnofsky Performance score. *Only significant factors in the univariate log-rank test were included into multivariate cox-regression analysis.

**Figure 6:**
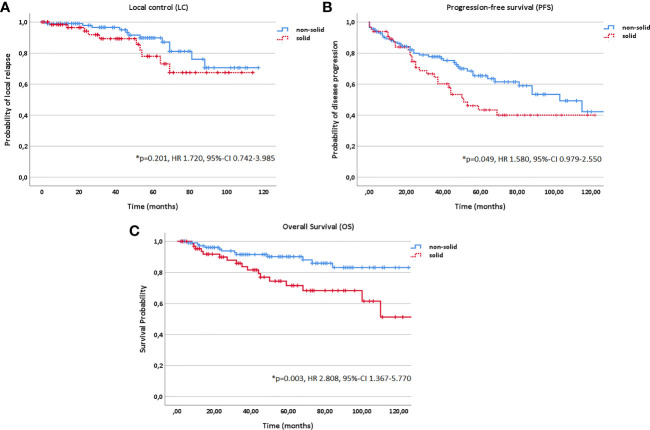
Kaplan-Meier estimates for local control **(A)**, progression-free survival **(B)** and overall survival **(C)** in dependence of histology (solid vs. non-solid).

In addition, age (10 year steps, HR 1.311, 95%-CI 1.134–1.517, *p<0.001*), KPS (<80% vs. ≥80%, HR 3.542, 95%-CI 2.010–6.240, *p<0.001*) and histology (solid vs. non-solid, HR 1.580, 95%-CI 0.979–2.550, *p=0.049*) correlated significantly with PFS in multivariate analysis; while, age (10 year steps, HR 1.963, 95%-CI 1.515–2.542, *p<0.001*), KPS (<80% vs. ≥80%, HR 6.472, 95%-CI 3.036–13.799, *p<0.001*), histology (solid vs. non-solid, HR 2.808, 95%-CI 1.367–5.770, *p=0.003*) and progression (yes vs. no, HR 89.553, 95%-CI 5.135–156.889, *p=0.002*) with OS (**Figures 2** and **6**).

### Toxicity Analysis

No interruptions of RT due to treatment-related side effects occurred. Overall, 39% of the patients in the definitive (n=12/31) and 44% of the patients in the postoperative bimodal RT group (n=77/176) claimed acute grade 2 and 3 toxicity. Predominantly, acute grade 2 and 3 dermatitis (35%, n=72/207), mucositis (33%, n=68/207), dys-/odynophagia (33%, n=68/207), dysgeusia/dysosmia (19%, n=39/207), xerostomia (17%, n=35/207) and decreased hearing (14%, n=29/207) were reported (overall 86% of all acute grade 2 and 3 side effects, n=114/132). At last follow-up, the majority of these symptoms had resolved (89%, n=117/132); only 11% of the patients who reported acute side effects grade 2 and 3 still suffered from a xerostomia (8%, n=11/132) or a persistant hearing deficit due to tympanic effusion grade 2 and 3 (3%, n=4/132).

Overall, grade 2 and 3 chronic toxicity was observed in 15% of the patients in the primary (n=5/31) and in 19% of the patients in the postoperative bimodal RT group (n=33/176). The major long-term grade 2 and 3 toxicities which occurred predominantly in postoperatively treated patients were xerostomia (5%, n=11/207), osteoradionecrosis (4%, n=8/207), persistant hearing deficits due to tympanic effusion (2%, n=4/207), brain barriers disorder of the temporal lobe (2%, n=4/207) and wound healing disorder (2%, n=4/207).

## Discussion

Given the rarity of ACC, the number of patients included in this study represents a considerably large collective of ACC of the major salivary glands (207 patients within the last 10 years) which were treated either with definitive (15%, n=31/207) or postoperative RT (85%, n=176/207). The primary aim of the current study was to describe long-term outcome of modern RT combining IMRT and CIRT and furthermore to assess prognostic factors for LC, PFS and OS for this regime.

Median follow-up was 50 months (range 3-121 months). For both definitive (15%, n=31/207) and postoperative RT (85%, n=176/207), excellent 10-year LC of 69% and OS of 73% were achieved in the current study. In comparison, 10-year LC and OS survival rates of 58% to 69% for LC and 49% to 65% for OS were reported for photon RT in comparable patient collectives (16, 27, 28). The superiority of the combined radiotherapy regime with IMRT and a carbon ion boost over photon RT for ACC was shown 2005 by Schulz-Ertner et al. in a prospective phase I/II pilot trial (29). The study included definitely treated patient as well as patients who received postoperative RT for R2 resection of the tumor. Overall, 63 patients were irradiated within the study (n=29 in the combined RT arm and n=34 in the photon RT arm). Study results showed beneficial outcome for the combined treatment regime with a 2-year LC rate of 72% vs. 25% and a 2-year OS rate of 87% vs. 78%. The study results were updated 2015 by Jensen et al. and showed comparable results to the previously published results by Schulz-Ertner et al. (30). Significantly increased LC and OS rates for combined RT vs. photon RT were observed with a 5-year LC of 60% vs. 40% and a 5-year OS of 77% vs. 59%. Nevertheless, in the last decades, the development of new photon RT techniques, i.e., the volumetric modulated arc therapy with constant dose rate, has led to an improvement of PTV coverage and sparing of organs at risk in head and neck tumors compared to step and shoot IMRT (Quelle). The advantages and disadvantages of these new methods over CIRT have to be proved in further studies.

Nevertheless, PFS with a decreased 10-year PFS rate of 38% after combined RT with IMRT and CIRT still remains challenging. In multivariate analysis, progression (yes vs. no, *p=0.002*) in contrast to local relapse (yes vs. no, *p=0.219*) negatively affected OS. It is known that the high rate of distant relapses in ACC patients limits survival significantly and requires new systemic treatment options. Although several systemic agents were investigated in the primary setting, the most effective systemic treatment still remains the CAP chemotherapy, which was established in the 1980s (31–33).

Two prognostic subgroups were identified in multivariate analysis: one subgroup resulting in decreased LC, PFS and OS rates and another subgroup having an additional survival disadvantage in PFS and OS. Patients with a macroscopic tumor disease (yes vs. no; *p<0.001* for LC, *p=0.010* for PFS and *p=0.040* for OS) treated in a definitive setting (vs. postoperative setting; *p=0.001* for LC, *p=0.006* for PFS, *p=0.049* for OS) and tumors of upper T stage (T1-4; *p=0.004* for LC, *p<0.001* for PFS, *p<0.001* for OS) showed significantly more local relapses and a decreased PFS and OS. Upper Age (*p<0.001* for both PFS and OS), lower Karnofsky Performance Score (<80% vs. ≥80%; *p<0.001* for both PFS and OS) and solid histology (vs. non-solid; *p=0.049* for PFS and *p=0.003* for OS) were in addition associated with worse survival outcome.

In the current study, patients profited significantly from postoperative vs. definitive RT regarding LC (*p<0.001*) and OS (*p=0.049*) with a 10-year LC and OS of 75% and 76% for postoperative RT and 49% and 61% for definitive RT while superior LC and OS could be achieved for definitive bimodal RT compared with definitive photon data (16, 22, 34–36). Although no randomized studies and prospective trials comparing treatment outcome of surgery alone vs. surgery in combination with postoperative RT are available, postoperative RT for resectable ACCs is considered as treatment of choice. Many retrospective studies have reported improved LC rates for postoperative RT compared to surgery alone, e.g., a study by Chen et al. from the University of California at San Francisco (11). The authors showed postoperative RT to be an independent prognostic factor for improved LC compared with surgery alone for 140 ACC patients (n=90 for postoperative RT, n=50 for surgery alone). For postoperative vs. definitive RT as well, no evidence-based guidelines exist. Nevertheless, in many retrospective studies, the impact of RT setting (postoperative vs. definitive RT) on LC and survival was discussed (16, 37, 38). 10-year OS rates of 42% for definitive RT and 55% for postoperative RT and 10-year LC rates of 56% for definitive RT and 91% for postoperative RT were reported by Mendenhall et al. for 101 patients with ACC of the minor and major salivary glands (16). Overall, 10-year LC rates for photon RT range between 36% and 43% for definitive RT (64 Gy to 70 Gy), between 61% and 79% for surgery alone and between 72% and 91% for postoperative RT (60 Gy to 72 Gy). Regarding OS, a 10-year OS rate of 37% to 42% for definitive photon RT, 59% to 68% for surgery alone and 55% to 65% for postoperative photon RT are reported (16, 22, 34–36). However, care must be taken when interpreting these results due to negative patient selection bias for definitive RT (inoperable tumors, patients with comorbidity, significantly more T3/4 and M1 stage and larger tumor volumes (**Table 1**)).

While positive resection margins (R1/2) were identified as independent negative prognostic factor particularly for LC by many authors, this could not be confirmed in the current study (*p=0.880*) (1, 11, 16, 20, 28). In a prospective phase-II trial, Jensen et al. as well could not identify any impact of resection status on LC (39). In contrary, the presence of a macroscopic tumor before RT start showed a significantly negative impact on all three time-to event data LC (*p<0.001*), PFS (*p=0.010*) and OS (*p=0.040*). Therefore, the MRIs for treatment planning performed in every patient before RT start were systematically evaluated. In addition, T stage had a significant impact on LC (p=0.004), PFS (p<0.001) and OS (p<0.001). In correlation to our results, a decreasing 5-year LC for upper T stage with a 5-year LC rate of 100% for T1, 80.2% for T2, 72.5% for T3, 70.9% for T4a and 38.6% for T4b were reported for CIRT by other authors (40). For photon beam RT, 5-year LC rates of 38% to 64% for T3 stage tumors and 14% to 44% for T4 stage tumors were observed (1, 11, 13, 16, 21). In addition, several studies associated upper T stage besides upper age, lower KPS, N+ stage and solid histology with the development of distant metastases and worse prognosis (41).

High-linear energy transfer (LET) RT with carbon ions leads to a dose-escalation due to a higher relative biological effectiveness compared with photon beam RT. In addition, extremely sharp dose gradients for precise dose delivery can be formed by the active-raster scanning technique, as used in the current study, which guarantees maintaining sufficient dose coverage of the target volume while preserving critical organs at risk (42). Treatment was well tolerated without any grade 4 or 5 toxicities. Detailed toxicity analysis showed moderate side effects with 43% acute (n=89/207) and 18% late (n=38/207) grade 2 and 3 toxicities. Predominantly, dermatitis, mucositis, dys-/odynophagia, dysgeusia/dysosmia, xerostomia and decreased hearing were observed during RT up to six weeks after RT. Regarding late toxicity, xerostomia, osteoradionecrosis, persistent hearing deficits due to tympanic effusion, brain barrier disorders of the temporal lobe and wound healing disorder were the most frequently reported side effects. In several studies, similar toxicity profiles for both photon RT and carbon ion RT are reported (21, 29, 39, 43). Although high RT doses (median total EQD2 of 80 Gy) were delivered in the current study, no grade 3 cranial nerve paralyses or visual impairments occurred. Osteoradionecrosis (4%, n=8/207) and brain barriers disorder (2%, n=4/207) were relatively rare as several authors described a dose-dependence between the probability for these symptoms and higher RT doses >70 Gy (44–49).

The limitations of the current study are predominantly methodological. The retrospective data collection from patient records leads possibly to incomplete or inaccurate data acquisition resulting in selection and information bias. That is why results should be considered critically under this aspect by the reader. In addition, due to the lack of prospective studies on ACC international treatment recommendations are lacking. As an example, the indication for postoperative RT differs in each center. In some centers, only patients with tumors in advanced stages or incomplete resection margins are irradiated postoperatively while in other centers every ACC patient receives postoperative RT regardless of T, N and R stage. The variety of treatment planning concepts, RT beam modalities (carbon ion RT, photon RT, proton RT), RT techniques (conventional RT, IMRT, passive raster scanning, active raster scanning) and dose calculation models for CIRT (two different models, e.g., Japan vs. Germany) make a clear comparison of the treatment results from differing centers difficult. In addition, different target volume definitions lead to different interpretations of local and regional relapses between the individual clinics. While some only count recurrences within the high risk volume (CTV1) as local recurrence, others regard local recurrences as failure within the low risk volume (CTV2). Although results of combined RT with IMRT and carbon ion RT are very promising, the availability of carbon ions is limited to a few centers worldwide and represents an expensive treatment option compared to photon beam RT (50).

## Conclusions

This current large German multicenter study shows that RT in a bimodal setting including IMRT and carbon ion boost for dose-escalation results in adequate LC in ACC of the major salivary glands with moderate toxicity. In multivariate analysis, upper T stage, macroscopic tumor resulted in significantly decreased LC rates. The high rate of T3/4 stages in the definitive RT group compared to the postoperative RT group makes the interpretation of the beneficial results in LC for postoperative RT difficult. Therefore, randomized trials are required.

## Data Availability Statement

The raw data supporting the conclusions of this article will be made available by the authors, without undue reservation.

## Ethics Statement

The studies involving human participants were reviewed and approved by the ethics committee of the University of Heidelberg, Germany (S421/2015). The patients/participants provided their written informed consent to participate in this study.

## Author Contributions

SAk and TB developed and planned the retrospective analysis. SAk is responsible for statistical considerations/basis of the analysis. All authors participated in data collection and/or interpretation of the results. SAk wrote the manuscript. All authors contributed to the article and approved the submitted version.

## Conflict of Interest

The authors declare that the research was conducted in the absence of any commercial or financial relationships that could be construed as a potential conflict of interest.

## Supplementary Material

Supplementary Table 1 Normal tissue constraints applied in the current study.
