# Supplementary material for: Large German Multicenter Experience on the Treatment Outcome of 207 Patients With Adenoid Cystic Carcinoma of the Major Salivary Glands
Source: Front Oncol. 2020 Nov 11;10:593379. doi: 10.3389/fonc.2020.593379 (PMC7686540; doi:10.3389/fonc.2020.593379)
Supplement: Supplementary Table 1 — Normal tissue constraints applied in the current study. [file Table_1.docx]

| **Suppl. table 1. Normal tissue constraints applied in the current study.** | |
| --- | --- |
| **Organ** | **Constraint** |
| Spinal cord | max 45 Gy |
| Chiasm/Optic nerves | max 54 Gy |
| Brainstem | max <54 Gy |
| Eyes (globe) | mean <30 Gy, max 54 Gy |
| Lens | max 7 Gy |
| Inner ear/Cochlea | mean <45 Gy |
| Parotid glands | mean <26 Gy |
| Submandibular glands | mean <35 Gy |
| Mandible | max 60 Gy |
| Oral cavity | mean <30 Gy, max <60 Gy |
| Larynx | mean < 45 Gy, max <66 Gy |
| **Abbreviations:** Gy= Gray, max=maximum. | |
